# Photon-Counting CT in Musculoskeletal Radiology: Technical Principles, Clinical Applications, and Future Directions

**DOI:** 10.5334/jbsr.4190

**Published:** 2026-02-20

**Authors:** Stijn De Bondt, Ione Limantoro, Hilde Bosmans, Geert Maleux, Nathalie Noppe, Michiel Herteleer

**Affiliations:** 1 Department of Radiology, UZ Leuven, Leuven, Belgium; 2 Department of Traumatology, UZ Leuven, Leuven, Belgium

**Keywords:** photon-counting CT, musculoskeletal radiology, CT imaging, spectral CT imaging

## Abstract

Photon-counting computed tomography is the newest technological advancement in CT imaging allowing for an improved spatial resolution, inherent spectral information, and significant radiation dose reduction. These benefits may enhance diagnostic confidence and accuracy for radiologists and clinicians. Multiple clinical applications in musculoskeletal radiology benefit from this technology, including the reduction of metal artifacts, improved visualization of bone marrow edema, and better assessment of crystal arthropathies. This narrative review presents a comprehensive summary of the current advancements and applications of this emerging technology within musculoskeletal radiology using several illustrative cases.

## Introduction

Computed tomography (CT) is a widely used imaging modality for the evaluation of various musculoskeletal pathologies. Since the introduction of the first CT-scanner into clinical practice, there has been tremendous technological progress in CT imaging. Both image acquisition and post-processing techniques have improved drastically [1].

In 2021, a major technological breakthrough in CT imaging entered clinical practice with the introduction of the first photon-counting CT (PCCT) scanner. Its novel detector design enables higher spatial resolution and intrinsic spectral information at an overall lower radiation dose [2].

In this narrative review, we summarize the technological differences in detector design and image acquisition, the clinical applications, and future directions of PCCT in musculoskeletal radiology based on the most recent literature.

### Differences in detector design of a photon-counting detector vs energy-integrating detector

**Figure 1:**
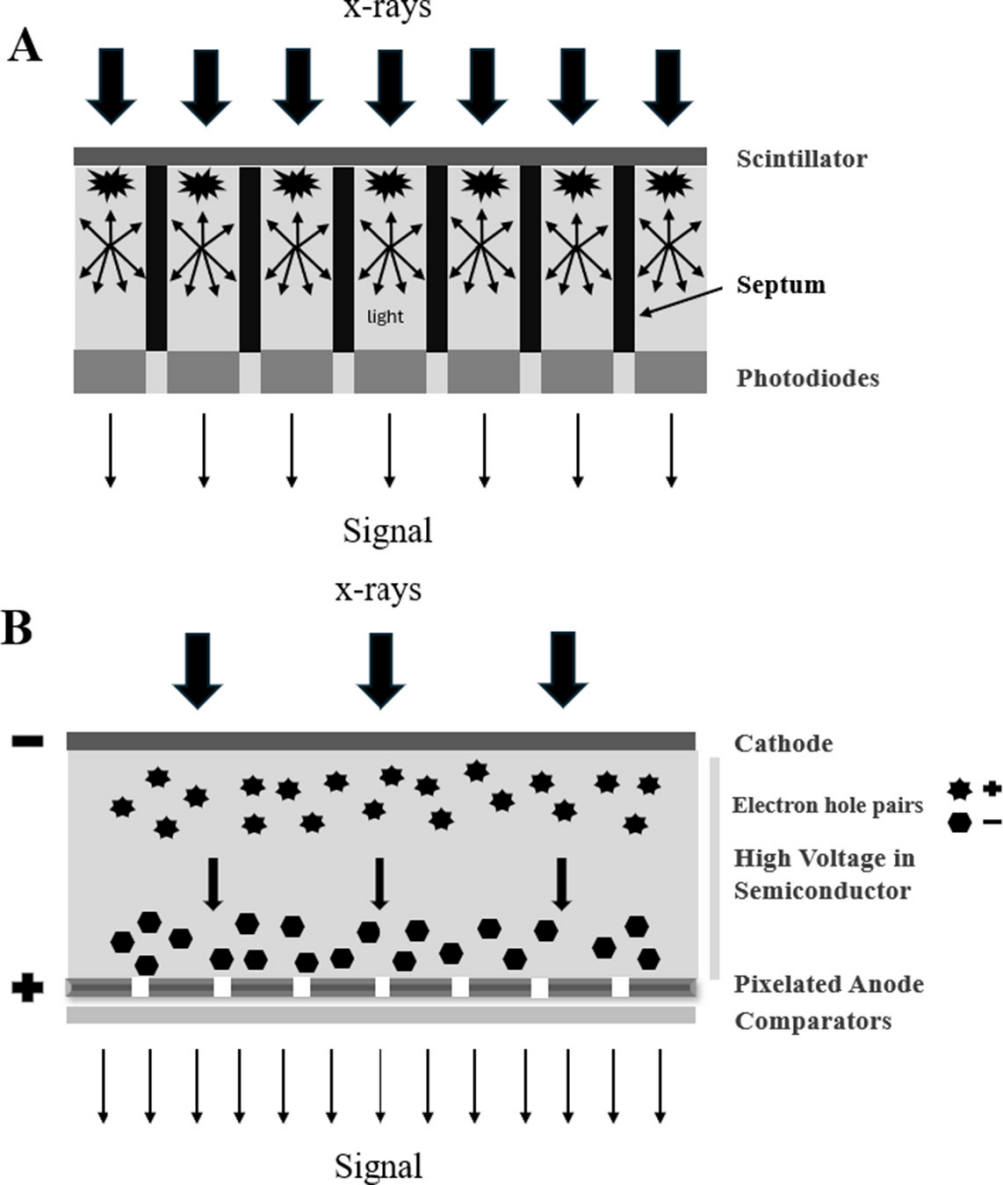
Schematic representation of the detector design of an energy integrating detector CT **(A)** and a photon-counting CT **(B)**.

Understanding the technical differences between the conventional energy-integrating detector (EID) and the photon-counting detector (PCD) is essential to understand the additional value of PCCT. These differences in detector design directly explain the superior imaging performance. Figure 1 is a schematic representation of both detector designs discussed below.

A conventional EID converts incoming X-ray photons into visible light photons using a scintillator layer. Optical septa prevent light spread between neighboring detector elements. The visible light photons are subsequently converted into an electronic signal by a photodiode array made of semiconducting material. The amplitude of the electrical signal is proportional to the total energy deposited on the photodiode. Lastly, the electrical signal is converted to a digital signal. However, the X-ray photons absorbed at the septa do not contribute to the signal, creating dead spaces and reducing the dose efficiency and spatial resolution of the detector [2–6]. Figure 1A is a schematic representation of the EID design.

A PCD uses a semiconductor made of a layer of material with a high-effective atomic number, such as cadmium telluride or silicon, converting the incoming X-ray photons into an electron-hole pair. A high voltage is applied in the detector causing the electron-hole pair to separate. The electrons shift towards the pixelated anode at the bottom of the semiconductor layer, creating a pulse with a pulse height proportional to the energy of the absorbed X-ray photon. Pulses exceeding a certain energy threshold are counted by multiple electronic comparators. The comparators sort the incoming photons into different energy bins. Since the PCD does not use visible light, septa are not necessary, eliminating the dead spaces [2–8]. Furthermore, the response time of the PCD to the incoming X-rays is faster than the EID, allowing for counting of the individual photons [5]. Figure 1B is a schematic representation of the PCD design.

### Technical advantages of photon-counting CT: ultra-high resolution imaging, dose efficiency, and spectral imaging

The PCCT offers several technical advantages resulting from the difference in detector design and image acquisition, leading to improved spatial resolution in ultra-high resolution (UHR) imaging, enhanced dose efficiency, and intrinsic spectral capability.

#### Ultra-high resolution imaging and dose efficiency of PCCT

**Figure 2:**
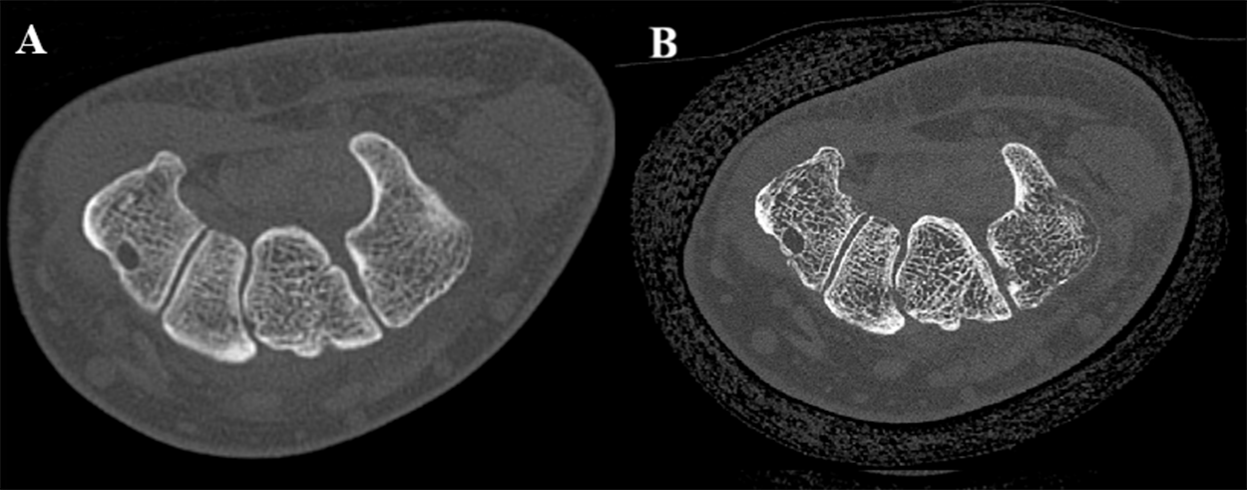
Post-traumatic CT imaging of the wrist in a 55-year-old male. **(A)** Initial post-trauma CT imaging performed on a Siemens SOMATOM Definition Flash CT. **(B)** Two months later, the patient was scanned on the Siemens NEAOTOM Alpha in ultra-high resolution enabling better depiction of bone microarchitecture at a significant lower radiation dose (CTDIvol of 10.4 mGy on PCCT compared to CTDIvol of 19.71 mGy on EID CT).

UHR CT imaging in PCCT is enabled by the absence of septa between detector elements, allowing for smaller detector elements. Pixel size in PCCT systems can be reduced to approximately 0.11 mm, significantly smaller than 0.25–0.50 mm in state-of-the-art EID systems [9]. Furthermore, in EID CT, UHR imaging requires the use of a comb filter which increases radiation dose and limits the field of view (FOV) [10, 11]. In PCCT systems, UHR imaging is possible without a comb filter thereby reducing the dose and FOV restrictions, allowing for UHR imaging of the trunk [10]. Additionally, a PCD filters out electronic noise caused by the electronic circuits in the detector by setting the energy threshold above the energy level of electronic noise, further improving the spatial resolution. This feature is especially beneficial in low-dose CT scans and in obese patients, where the signal is weak and the noise level is high [3–5]. Third, the high-energy X-rays are overweighted compared to low-energy X-rays in EID CT because the EID integrates the energy from all photons. In a PCD, each energy level of the photon is uniformly weighted which will improve contrast between different tissues [3–5]. Figure 2 highlights the improved spatial resolution of the appendicular skeleton at a significant radiation dose reduction.

Several studies compared UHR imaging of PCCT with EID CT. Rajendran et al. compared the UHR images of a PCCT to EID CT for the wrist in twelve patients and found a better visualization of bone structures at a radiation dose reduction of 49% [12]. Similar results were found in a prospective study on 32 patients by Baffour et al., demonstrating a superior visibility of anatomic structures in the shoulder and pelvis with a radiation dose reduction of 31–47% on UHR PCCT images compared to UHR EID CT images [13]. A cadaveric study by Patzer et al. demonstrated a superior trabecular microstructure delineation of the shoulder at the same radiation dose on PCCT compared to a dual-source EID CT [10]. Several other clinical or cadaveric studies showed similar results with an improved contrast-to-noise ratio (CNR) and visibility of bone microstructure at a significantly lower radiation dose [14–18].

These studies confirm the hypothesis of improved depiction of trabecular bone at a lower dose in PCCT compared to EID CT, which could increase the diagnostic accuracy in various bone pathologies.

#### Spectral imaging

Another major advantage of PCCT is its intrinsic spectral capability. Multiple comparators in the PCD count the number of pulses with an energy height greater than a preset threshold and sort them into energy bins, ranging from two to eight bins depending on the vendor [2–7]. As a result, multi-energy information is acquired with every scan without requiring dedicated dual-energy protocols or dose penalty. In EID CT, spectral information is not inherently available, but only after choosing a certain scan protocol on a dual-energy CT (DECT) scanner.

Spectral imaging enables different post-processing tools; for example, virtual monoenergetic imaging (VMI) or material decomposition images such as virtual non-calcium (VNCa) images. VNCa images are created by subtracting the calcium signal allowing for visualization of bone marrow edema (BME) or to identify uric acid crystals in gout [19].

These technological characteristics translate into several clinical advantages in musculoskeletal imaging, as discussed below.

### Clinical applications

The technological strengths of a PCCT system permit several clinical applications improving the diagnostic performance of musculoskeletal CT imaging. Several post-processing techniques demonstrate a promising potential to improve diagnostic accuracy across a range of clinical applications. In the following paragraphs, these clinical applications will be discussed with illustrative cases from the University Hospitals of Leuven on the Siemens NAEOTOM Alpha. Table 1 summarizes the clinical applications.

**Table 1:** Summary of the clinical applications of PCCT imaging.

APPLICATION	CLINICAL CONTEXT	ADVANTAGES
**Musculoskeletal pediatric imaging**	Trauma, infection, congenital diseases, and bone/soft tissue tumors	Improved imaging at a significant dose reduction
**Metal artifact reduction**	Evaluation of prostheses/screws and periprosthetic fractures	Reduced artifacts enhancing diagnostic accuracy
**Bone marrow edema maps**	Trauma and insufficiency fractures	Inherent spectral data allows for visualization of bone marrow edema associated with fractures
**Crystal arthropathy**	Evaluation of crystal arthropathies	Improved detection and differentiation of crystal arthropathies
**Multiple myeloma**	Detection of osteolytic bone lesions	Significant radiation dose reduction and potential assessment of activity level of lesions
**Cartilage and subchondral bone imaging**	Osteoarthritis	Improved evaluation of cartilage and subtle subchondral lesions
**Bone mineral density**	Opportunistic osteoporosis screening and prediction of fracture risk	BMD measurement and fracture risk assessment on routine PCCT scans
**Soft tissue**	Evaluation of soft tissue injury	Possibility of evaluating soft tissue injury

#### Pediatric musculoskeletal imaging

**Figure 3:**
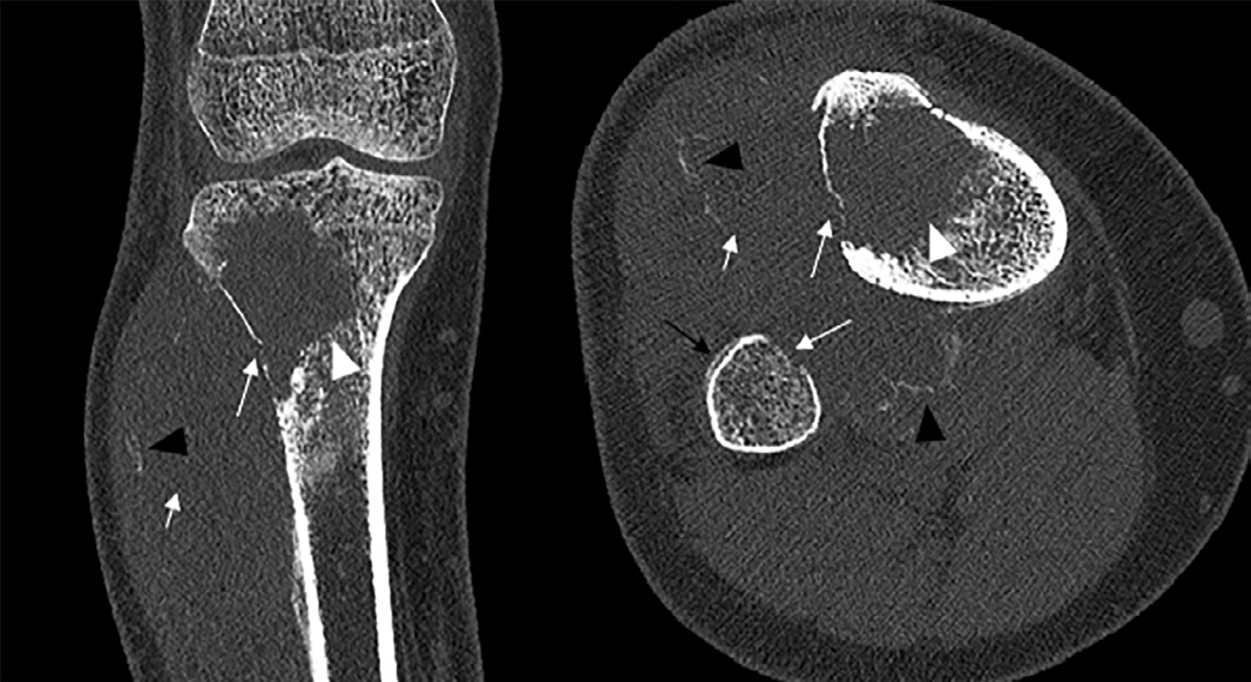
A 13-year-old female with an osteosarcoma in the right proximal tibia manifesting as an osteolytic lesion (white arrowhead) with cortical disruption of the lateral wall of the proximal tibia and the medial wall of the proximal fibula (long white arrow) and a notable extra-ossous component invading the anterior muscle compartment (short white arrow) with mineralization in the soft tissue component (black arrowhead). Furthermore, a subtle periosteal reaction at the anterior wall of the proximal fibula is noted (black arrow).

In children, CT imaging is performed for the diagnosis or evaluation of a variety of musculoskeletal pathologies, including trauma, bone or soft tissue tumors, or osteomyelitis. Especially in the pediatric population, dose efficiency of the PCCT is beneficial. Studies have shown that the use of PCCT results in reduced radiation dose while maintaining or even increasing image quality in children, for example, in temporal bone imaging and chest imaging [20, 21]. In musculoskeletal imaging, improved spatial resolution using thin slices and sharp reconstruction kernels allows for better visualization of cortical bone and trabecular structures, enabling immaculate delineation of bone pathology (Figure 3) [13, 22–25]. PCCT could also improve assessment of complications in pediatric orthopedic surgery using metal artifact reduction techniques [26]. Additional spectral images, such as BME maps, could further improve diagnostic accuracy. Currently, studies regarding pediatric musculoskeletal PCCT imaging are still limited.

#### Metal artifact reduction

Visualizing subtle loosening of prostheses or screws, implant fractures, and periprosthetic fractures remains challenging in CT imaging due to metal artifacts. Dense objects cause selective attenuation of low-energy photons in the multi-energetic X-ray beam, causing an increase in the mean energy of the X-rays. This phenomenon is known as beam hardening and causes characteristic streaking artifacts. The high attenuating property of dense objects also results in ‘photon starvation’ whereby fewer photons reach the detector causing missing information which further increases artifacts [3, 27]. In a PCCT, a constant weighting of photons reduces the artifacts associated with beam hardening [3].

**Figure 4:**
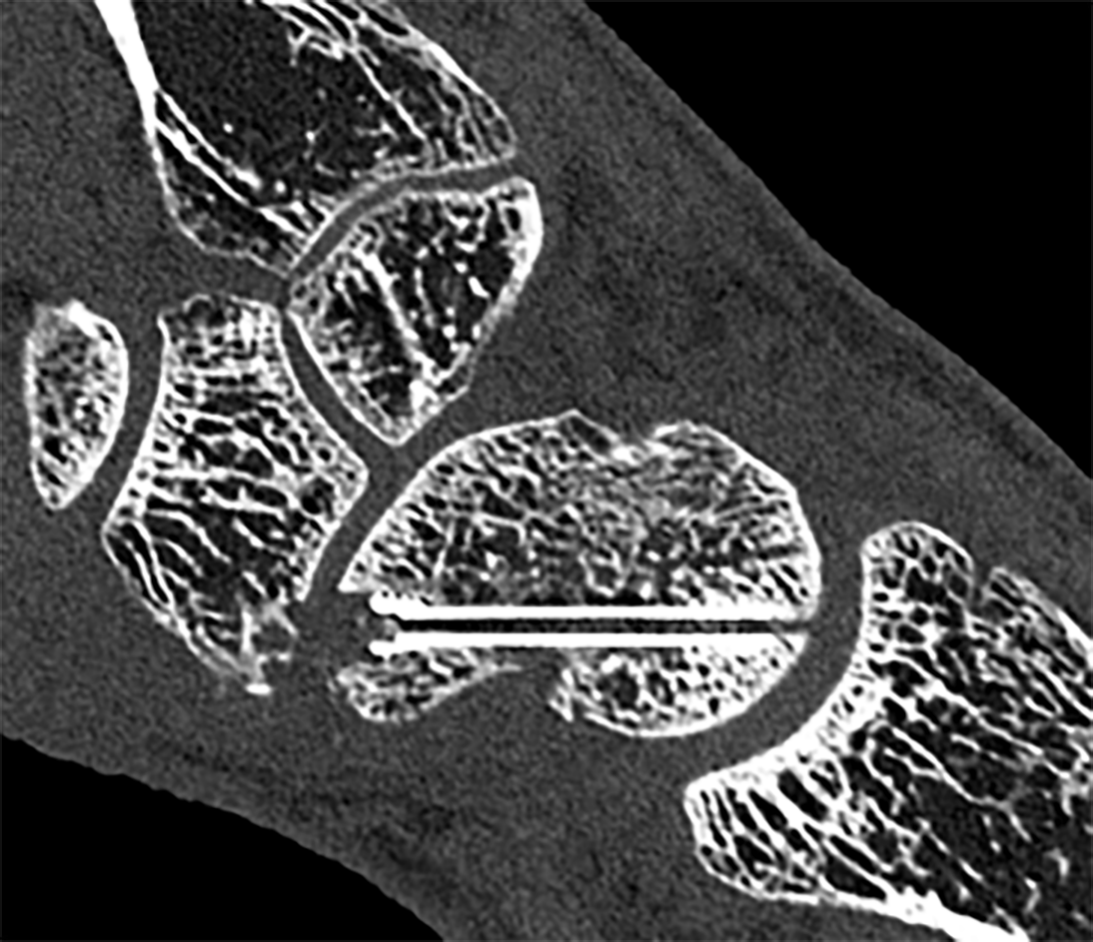
PCCT image post scaphoid screw fixation for a scaphoid fracture in a 45-year-old male with excellent metal artifact reduction using tin filtration and IMAR, allowing for visualization of trabecular structure in the thread of the screw at a CTDIvol of 15.5 mGy.

**Figure 5:**
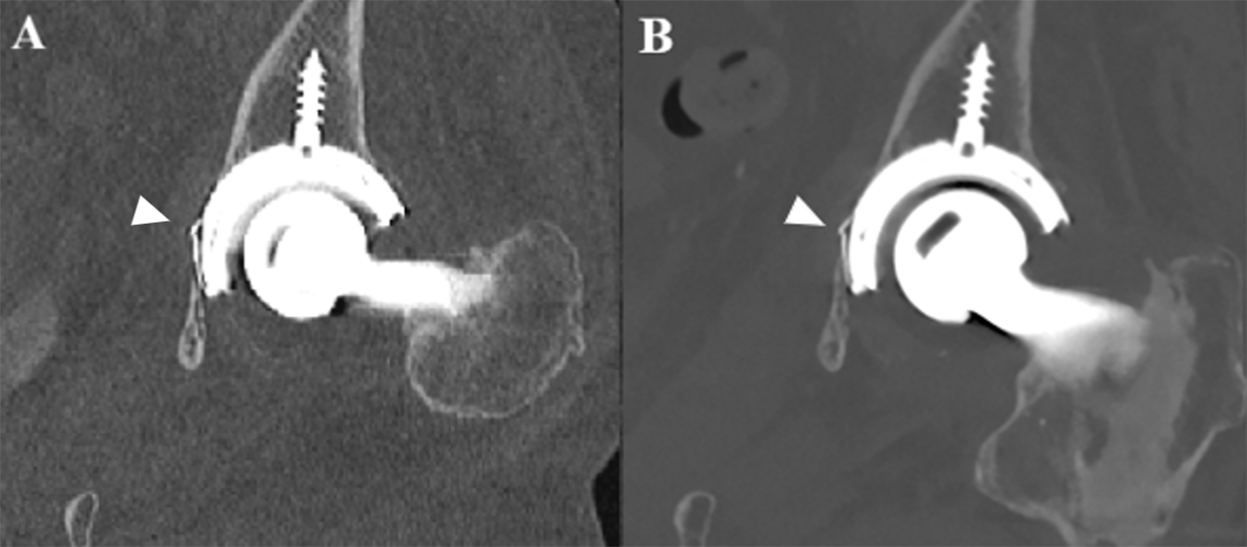
An 81-year-old female consulting the emergency department after trauma with an implant fracture of the acetabular cup of the hip prosthesis. **(A)** Coronal slice with IMAR showing an implant fracture (white arrowhead) on a Siemens SOMATOM Definition Flash. **(B)** An additional IMAR PCCT scan was performed on the Siemens NEAOTOM Alpha to enhance characterization of the implant fracture (white arrowhead) highlighting the improved spatial resolution and metal artifact reduction at a lower radiation dose compared to EID CT (CTDIvol of 11.8 mGy vs 13.3 mGy).

Several techniques have been introduced to limit metal artifacts. First, PCCT also uses iterative metal artifact reduction (IMAR) reconstruction technique to reduce artifacts of dense objects [28]. Second, since the introduction of spectral CT imaging (DECT and PCCT), VMI has been instrumental for several clinical applications, including further reduction of metal artifacts [29–32]. VMI images are a result of post-processing spectral CT images in which images of a single energy level (keV) are reconstructed [33]. Multiple studies suggested the use of VMI images at >100 keV for optimal reduction of metal artifacts [29–32]. In a recent study, Schreck et al. demonstrated the utility of combining IMAR with VMI images at keV levels >100 keV for improved reduction of artifacts of dental implants [34]. Third, Marth et al. demonstrated a significant reduction in artifacts of ankle prosthesis by using tin filtration on PCCT [29]. Figures 4 and 5 showcase the metal artifact reduction capabilities of PCCT.

Based on available literature, optimal metal artifact reduction on PCCT can be achieved by using the combination of tin filtration, IMAR reconstruction, and VMI at >100 keV. However, further studies are needed to optimize patient- and material-specific PCCT scanning protocols and the optimal keV value of VMI. The best keV value for reducing metal artifacts on VMI probably depends on multiple factors including the material and more specifically the type of implant/prosthesis/screw, manufacturer, body part, and body composition.

#### Skeletal trauma: fractures and bone marrow edema

Subtle fractures without significant cortical disruption and the extent of fracture lines can be challenging to evaluate on regular CT imaging. MRI is considered the gold standard in diagnosis of subtle fractures due to accurate visualization of BME resulting in a higher diagnostic accuracy in detecting occult fractures [35, 36]. However, most hospitals do not have an MRI available in the emergency department making CT the main diagnostic tool for post-traumatic fractures.

In 2007, the DECT was introduced allowing for material decomposition based on differences in X-ray absorption of tissue at low and high energy levels [37]. Post-processing of spectral imaging allows for VNCa and BME images which highlight BME associated with fractures [37]. Multiple studies on DECT demonstrated the benefits of BME maps in the diagnosis of (occult) fractures [37–41].

**Figure 6:**
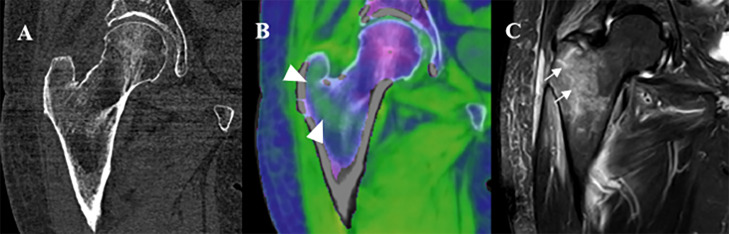
An 81-year-old woman consulted the emergency department with right hip pain after a low-energy trauma. **(A)** No clear fracture was visible on the grayscale images on the PCCT. **(B)** However, the bone marrow edema map shows intertrochanteric edema (white arrowheads), suggestive of a fracture. **(C)** MRI the following day confirms an intertrochanteric fracture (white arrows).

By combining UHR with spectral imaging and additional noise reduction, PCCT could generate BME maps with higher accuracy compared to DECT BME maps. This hypothesis was recently confirmed by a phantom study of Rajendran et al., demonstrating a higher accuracy for calcium quantification of a PCCT with tin filtration compared to DECT [42]. Furthermore, PCCT offers superior UHR imaging improving diagnostic confidence compared to EID CT, as demonstrated by Kämmerling et al. in scaphoid fractures [43]. Figure 6 highlights the added value of BME maps on PCCT in improving diagnostic accuracy of occult hip fractures. However, clinical studies on the sensitivity and specificity of PCCT BME images are still lacking.

#### Crystal arthropathy differentiation

Crystal arthropathies, including gout, calcium pyroph-osphate deposition disease (CPPD), and basic calcium phosphate (BCP) deposition diseases, are common [44]. Ultrasound is a commonly used imaging modality for gout; however, ultrasound is operator-dependent [45]. Material decomposition capability of spectral CT imaging allows for detection and potential characterization of different types of crystal arthropathies [45–47]. However, in 50% of patients with symptoms lasting less than 2 years monosodium urate (MSU) crystals were too small to detect on DECT, due to a lack of spatial resolution [45]. In gout, both ultrasound and DECT are the recommended imaging modalities for diagnosis, monitoring, and assessment of therapy response. In CPPD and BCP, ultrasound and conventional radiography remain the recommended imaging modality [45].

**Figure 7:**
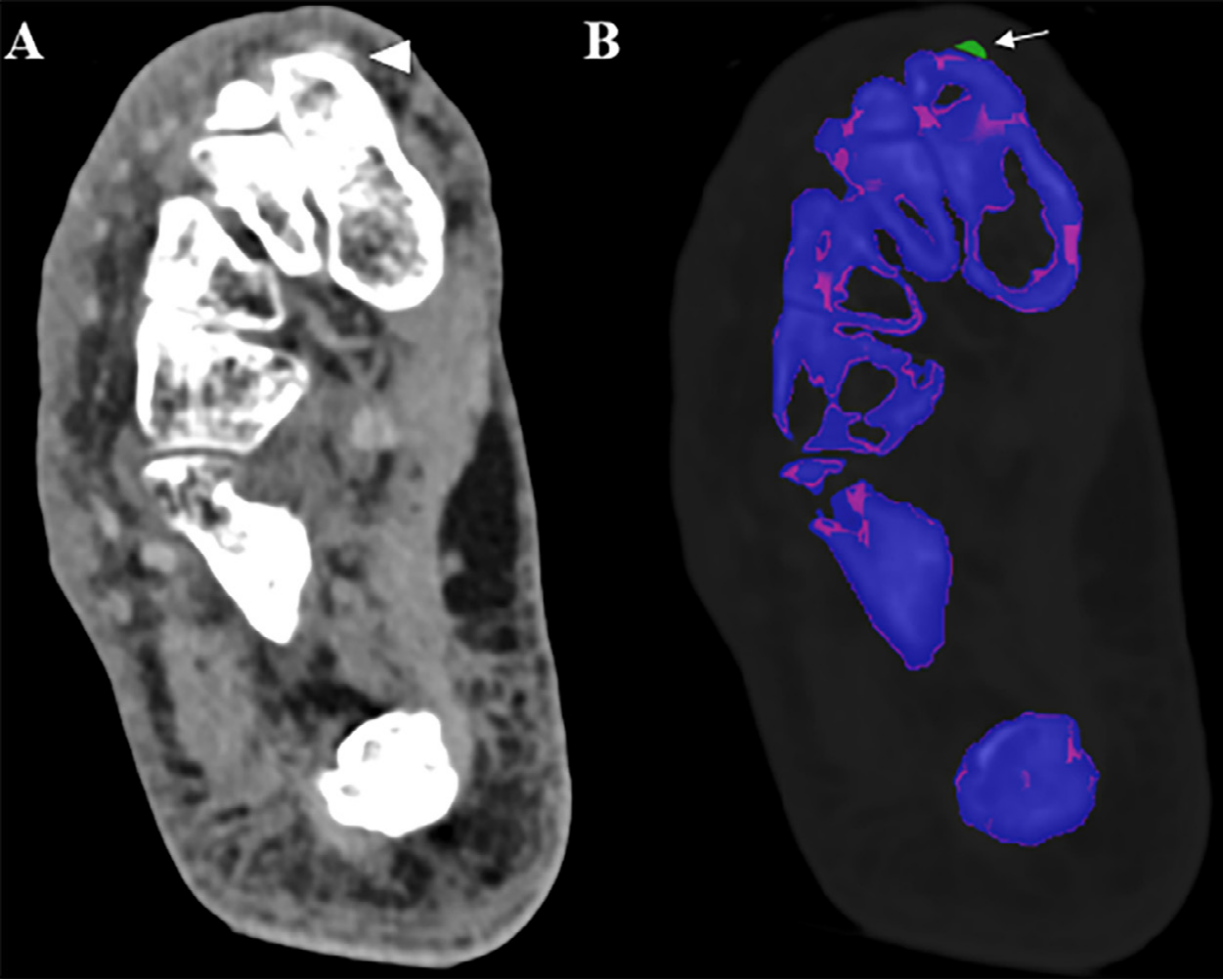
A 76-year-old female presenting with pain and redness in the forefoot. **(A)** A small dense area in the soft tissue surrounding the distal part of the medial cuneiform bone (white arrowhead) on the grayscale CT images, suggestive of gout. **(B)** Post-processing images using the gout application in SyngoVia highlight the monosodium urate crystals in green (white arrow).

Improved spatial resolution and spectral capabilities of PCCT are promising in detection and differentiation of crystal arthropathy (Figure 7). Multiple in vitro studies have demonstrated that PCCT can differentiate MSU, CPP, and hydroxyapatite (HA) crystals with improved spatial resolution compared to DECT [48–51]. Larger clinical studies are warranted to validate these results in a clinical setting.

#### Multiple myeloma imaging

Multiple myeloma (MM) is associated with characteristic ‘punched out’ osteolytic bone lesions dispersed in the axial and appendicular skeleton [52]. Low-dose whole-body CT, whole-body MRI, or positron emission tomography (PET) imaging can be used in the diagnosis and treatment monitoring of osteolytic bone lesions of MM. Lately, PET-CT has been proposed as the first-choice imaging modality allowing for assessment of the activity level of the bone lesions [53].

However, the introduction of ultra-low dose PCCT imaging with improved spatial resolution and inherent spectral information could prove useful in MM diagnosis and follow-up. A recent study by Heidemeier et al. found a significant difference in attenuation on VMI images at different keV levels and VNCa images across patients with therapy response versus initial diagnosis or disease progression [54]. Subsequently, multiple studies on MM have demonstrated an improved image quality at reduced radiation dose on PCCT [55–58]. Further studies should be conducted to assess the added benefits of spectral PCCT imaging in the evaluation of the activity level of the osteolytic bone lesions.

### Osteoarthritis: cartilage visualization and arthrography

**Figure 8:**
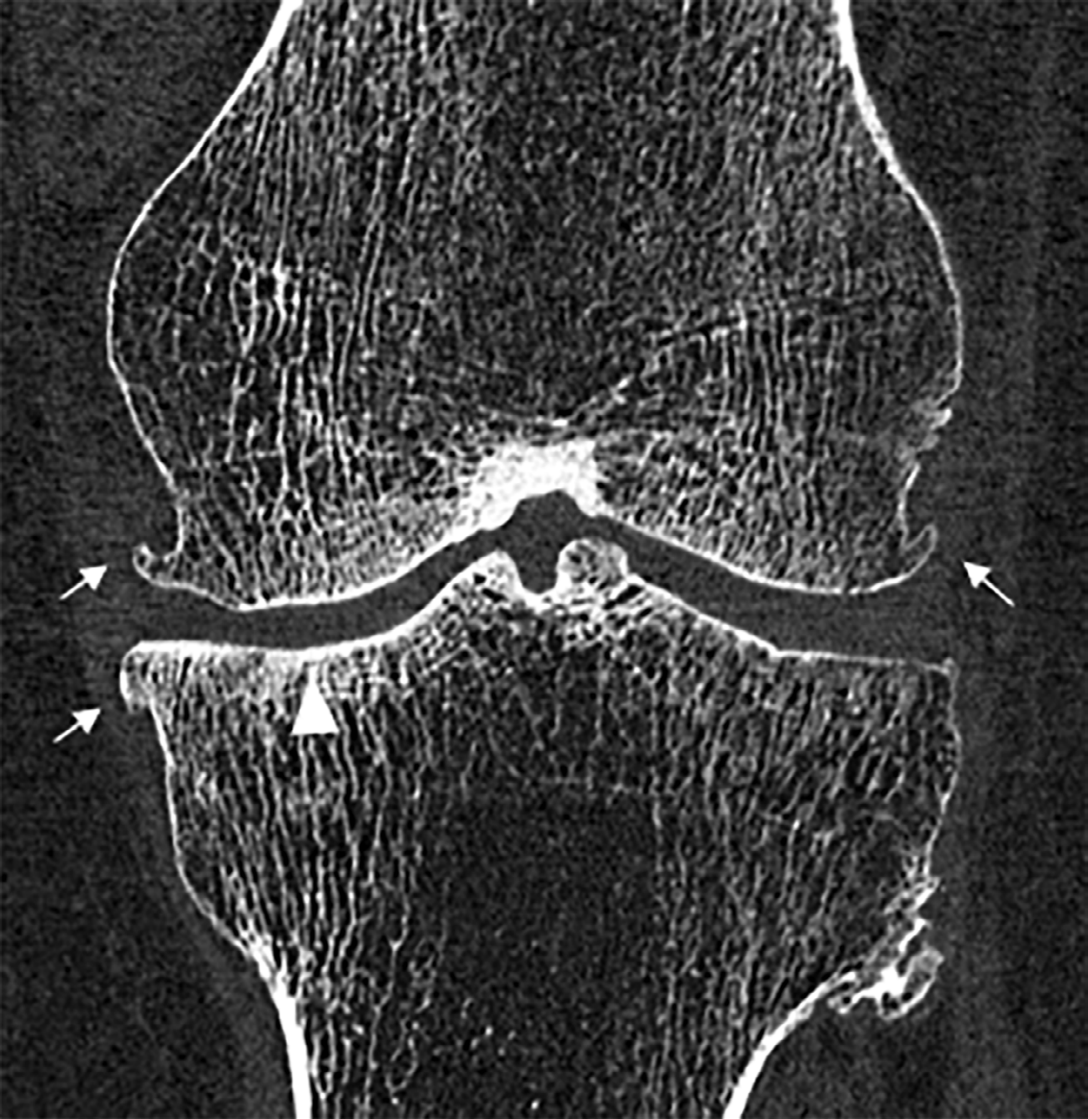
Ultra-high resolution (UHR) PCCT scan in a 74-year-old woman with osteoarthritis of the knee. The UHR allows for meticulous depiction of the trabecular structure as well as the secondary bone remodeling with sclerosis of the medial tibial condyle (white arrowhead) and osteophyte formation bilaterally (white arrows).

Osteoarthritis affects 10–18% of elderly people worldwide, for which MRI, with or without intra-articular contrast injection, is the most sensitive imaging modality [59]. However, CT is more readily available and has less contraindications. Improved spatial resolution on PCCT allows for immaculate visualization of secondary bone remodeling associated with osteoarthritis (Figure 8). Furthermore, PCCT might be suitable to evaluate the cartilage. A cadaveric study by Garcelon et al. showed that PCCT enabled delineation of cartilage of the knee without using an intra-articular contrast agent [60]. Another cadaveric study of the knee by Chappard et al. evaluated the use of VMI for visualization of cartilage and bone and found an optimal keV values of 60 and 70, allowing for simultaneous evaluation of bone and cartilage integrity [61]. Validation studies in patients are still lacking.

CT arthrography is the most accurate imaging modality in evaluating cartilage thickness and lesions associated with osteoarthritis [62]. UHR PCCT arthrography offers stellar image quality improving conspicuity of anatomical structures, as shown by Luetkens et al. [63]. A recent cadaveric study by de Boer et al. found a similar image quality at lower total radiation dose of PCCT arthrography of the wrist compared to EID CT [64]. PCCT could therefore be instrumental in detection of early signs of osteoarthritis as well as improve diagnostic confidence.

#### Bone mineral density

Osteoporosis is highly prevalent in the elderly population with associated fractures at low-energy trauma [65]. Osteoporosis is caused by a misbalance in bone remodeling with a greater bone resorption than formation [66]. Bone mineral density (BMD) is used to quantify the level of osteopenia and osteoporosis. As of today, dual-energy X-ray absorptiometry (DXA) is the gold standard in measuring the BMD and assessment of the fracture risk associated with osteoporosis [66]. However, several approaches for opportunistic BMD measurements on CT imaging have been described but these methods are generally imprecise [67]. Several cadaveric studies found that PCCT can estimate bone strength, BMD, and bone turnover with good accuracy [68, 69]. Furthermore, recent clinical studies by El Sadaney et al. and Zhou et al. used PCCT spectral localizer images of lumbar PCCT scans to derive BMD values with associated T-scores and found a high diagnostic performance for osteoporosis [67, 70]. These studies highlight the possibility of opportunistic osteoporosis diagnosis on the available scout view of (lumbar) PCCT.

#### Soft tissue injury

Trauma is frequently associated with soft tissue injury consisting of ligamentous or muscle injury for which MRI is the gold standard imaging modality [71, 72]. Yet, MRI is not readily available in most trauma departments. PCCT allows for improved soft tissue contrasts combined with the intrinsic spectral information in UHR which could transform diagnostic accuracy of soft tissue injuries on CT [73]. Zijta et al. reported on the feasibility of the simultaneous evaluation of fractures, bone marrow lesions, and soft tissue injury of acute knee trauma using a dual-source PCCT [74]. However, prospective clinical studies comparing diagnostic performance of PCCT to MRI are still lacking.

### Future directions of PCCT in musculoskeletal radiology

PCCT remains a new advancement in CT imaging. As of today, large clinical studies on the diagnostic capabilities of the PCCT for most musculoskeletal applications are yet to be conducted. Furthermore, scanning protocols should be optimized for each clinical application to maximize diagnostic performance and dose efficiency. However, the initial research shows promising results.

Since its introduction in 2012, radiomics has gained traction to aid physicians in clinical decision making. Radiomics is a quantitative analysis of many medical imaging features of tumors, including prognosis and genomics, offering information on cancer phenotype and prognosis [75, 76]. Radiomics has also been used to identify osteoporosis [75]. A phantom study by Hertel et al. showed a high stability of a substantial portion of radiomics features on PCCT [77]. Improved spatial resolution and spectral information of PCCT could prove instrumental for enhancing tumor diagnosis using radiomics. Further studies with clinical PCCT images are warranted.

The improved spatial resolution and the spectral information of PCCT images could also be leveraged for optimizing artificial intelligence tools which could improve diagnostic accuracy of radiologists. UHR imaging with PCCT could also enhance the accuracy of the Bone Strength (BOS) score measurement, which is used for predicting pathological fracture risk in the femur through patient-specific finite element analysis. The BOS score supports orthopedic surgeons and oncologists in selecting the optimal treatment option in femoral bone metastasis [78].

New developments may take place based on what will be learned in the near future in all these studies.

## Conclusion

PCCT is a major leap forward in CT imaging. Its new detector design enables improved spatial resolution, substantial radiation dose reduction, and intrinsic spectral capabilities. Initial research in musculoskeletal applications of the PCCT shows promising results; however, large clinical studies are needed to determine the optimal settings for different indications and validate its diagnostic and clinical impact.
